# Rabies

**Published:** 1896-09

**Authors:** 


### EDITORIAL.

THE Philadelphia branch of the American Antivivisection Society is out with two timely monographs bearing upon the subject of rabies and hydrophobia and on mimetic diseases. The variations of hydrophobia are well shown by statistical references and the practically unanimous report of a large number of prominent members of the medical profession who have given the subject special study and investigation for a long period of years. The many other diseases with similar groups of symptoms closely allied to those said to be diagnostic of hydrophobia are lucidly referred to, and the influence of fear and mental anxiety—lyssophobia—are forcibly presented. The great injury done by newspaper reports is thoroughly denounced and charged with the responsibility of a large proportion of the many so-called cases. Veterinarians who have to deal directly with rabid dogs have long been aware of the rarity of hydrophobia, and as a profession are strangers to its existence themselves, though often exposed to its alleged dangers. The writer for years has adopted a policy of impressing upon all connected with the home or ownership of dogs affected with conditions considered to be rabies an ignoring of all danger associated with prior contact with these animals, and in a district from which during the past ten years he has taken out ten or more animals so suffering there has not been a single reported case of hydrophobia. In every instance he has been able to deny the existence of this disease among dogs to reporters, with the results to the human family as above cited.

